# Comparison of resistive switching characteristics using copper and aluminum electrodes on GeO_x_/W cross-point memories

**DOI:** 10.1186/1556-276X-8-509

**Published:** 2013-12-05

**Authors:** Sheikh Ziaur Rahaman, Siddheswar Maikap

**Affiliations:** 1 Thin Film Nanotech Lab., Department of Electronic Engineering, Chang Gung University, 259 Wen-Hwa 1st Rd., Kwei-Shan, Tao-Yuan 333, Taiwan

**Keywords:** Memory, Resistive switches, GeO_*x*_, Copper, Aluminum, Solid electrolyte

## Abstract

Comparison of resistive switching memory characteristics using copper (Cu) and aluminum (Al) electrodes on GeO_*x*_/W cross-points has been reported under low current compliances (CCs) of 1 nA to 50 μA. The cross-point memory devices are observed by high-resolution transmission electron microscopy (HRTEM). Improved memory characteristics are observed for the Cu/GeO_*x*_/W structures as compared to the Al/GeO_*x*_/W cross-points owing to AlO_*x*_ formation at the Al/GeO_*x*_ interface. The RESET current increases with the increase of the CCs varying from 1 nA to 50 μA for the Cu electrode devices, while the RESET current is high (>1 mA) and independent of CCs varying from 1 nA to 500 μA for the Al electrode devices. An extra formation voltage is needed for the Al/GeO_*x*_/W devices, while a low operation voltage of ±2 V is needed for the Cu/GeO_*x*_/W cross-point devices. Repeatable bipolar resistive switching characteristics of the Cu/GeO_*x*_/W cross-point memory devices are observed with CC varying from 1 nA to 50 μA, and unipolar resistive switching is observed with CC >100 μA. High resistance ratios of 10^2^ to 10^4^ for the bipolar mode (CCs of 1 nA to 50 μA) and approximately 10^8^ for the unipolar mode are obtained for the Cu/GeO_*x*_/W cross-points. In addition, repeatable switching cycles and data retention of 10^3^ s are observed under a low current of 1 nA for future low-power, high-density, nonvolatile, nanoscale memory applications.

## Background

Recently, resistive switching memory devices involving different materials such as Pr_0.7_Ca_0.3_MnO_3_ (PCMO) [1], NiO_*x*_[2], SrTiO_3_[3,4], TaO_*x*_[5-8], HfO_*x*_[9,10], TiO_2_[11], ZrO_2_[12], Na_0.5_Bi_0.5_TiO_3_[13], and AlO_*x*_[14-16] are widely reported to replace conventional flash memory. On the other hand, conductive bridging resistive random access memory (CBRAM) involving the migration of cations (Ag^+^ or Cu^z+^, *z* = 1, 2) in solid electrolytes such as Ge_*x*_Se_1-*x*_[17-20], GeS_2_[21], Ta_2_O_5_[22], ZrO_2_[23-25], TiO_*x*_/ZrO_2_[26], GeSe_*x*_/TaO_*x*_[27], HfO_2_[28], CuTe/Al_2_O_3_[29], Ti/TaO_*x*_[30], ZnO [31], SiO_2_[32], and GeO_*x*_[33] is also reported. In this case, the mobile Ag^+^ or Cu^z+^ ions play an important role in the formation and dissolution of metallic filament in the solid electrolytes. Although memory characteristics using different solid electrolytes have been reported, GeO_*x*_-based CBRAM devices in the cross-point structure are also a beneficial choice. Memory characteristics using GeO_*x*_ film in a Cu/GeO_*x*_/Al structure were first reported by Beynon and El-Samanoudy in 1987 [34]. Their extended work was published in 1991 using a Cu/GeO_*x*_/Au structure [35]. Resistive switching memory using GeO_*x*_ material in different structures such as Ni/GeO_*x*_/SrTiO_*x*_/TaN [36] and Pt/SiGeO_*x*_/SiGeON/TiN [37] has also been reported for future nonvolatile memory applications. On one hand, Schindler et al. [38] has reported a GeO_*x*_ layer for the Cu (Ag) diffusion barrier layer in a Cu (Ag)/GeSe/Pt structure. On the other hand, cross-point structures using different switching materials have been reported by several groups [6,39-42] to have a high-density memory for future applications. It is known that resistive switching memories in cross-point architecture possess several attractive features and have attracted considerable attention in recent years because of the multilayer stacking of three-dimensional (3D) architecture, simplicity of their manufacturing, and the simplest interconnection configuration. Furthermore, resistive switching memory devices with low-current operation (<100 μA) are also an important issue. To mitigate those specifications, a cross-point memory using a Cu/GeO_*x*_/W structure has been compared with that using an Al/GeO_*x*_/W structure for the first time.

In this study, the memory characteristics using Cu and Al top electrodes (TEs) on GeO_*x*_/W cross-points have been compared. The cross-point structures were observed by high-resolution transmission electron microscopy (HRTEM). The Cu/GeO_*x*_/W cross-point memory devices have shown improved bipolar resistive switching characteristics as compared to the Al/GeO_*x*_/W cross-points, owing to the AlO_*x*_ layer formation at the Al/GeO_*x*_ interface. The RESET current deceases with the decrease of current compliances (CCs) from 50 μA to 1 nA for the Cu/GeO_*x*_/W devices, while the RESET current was independent (>1 mA) of CC in the range of 500 μA to 1 nA for the Al/GeO_*x*_/W cross-point memories. High resistance ratios of 10^2^ to 10^4^ under bipolar and approximately 10^8^ under unipolar modes are observed for the Cu/GeO_*x*_/W cross-point memory devices. Repeatable switching cycles and data retention of approximately 10^3^ s under a low CC of 1 nA were obtained for the Cu TE devices, which are very useful for low-power operation of high-density nonvolatile nanoscale memory applications.

## Methods

**Figure 1:**
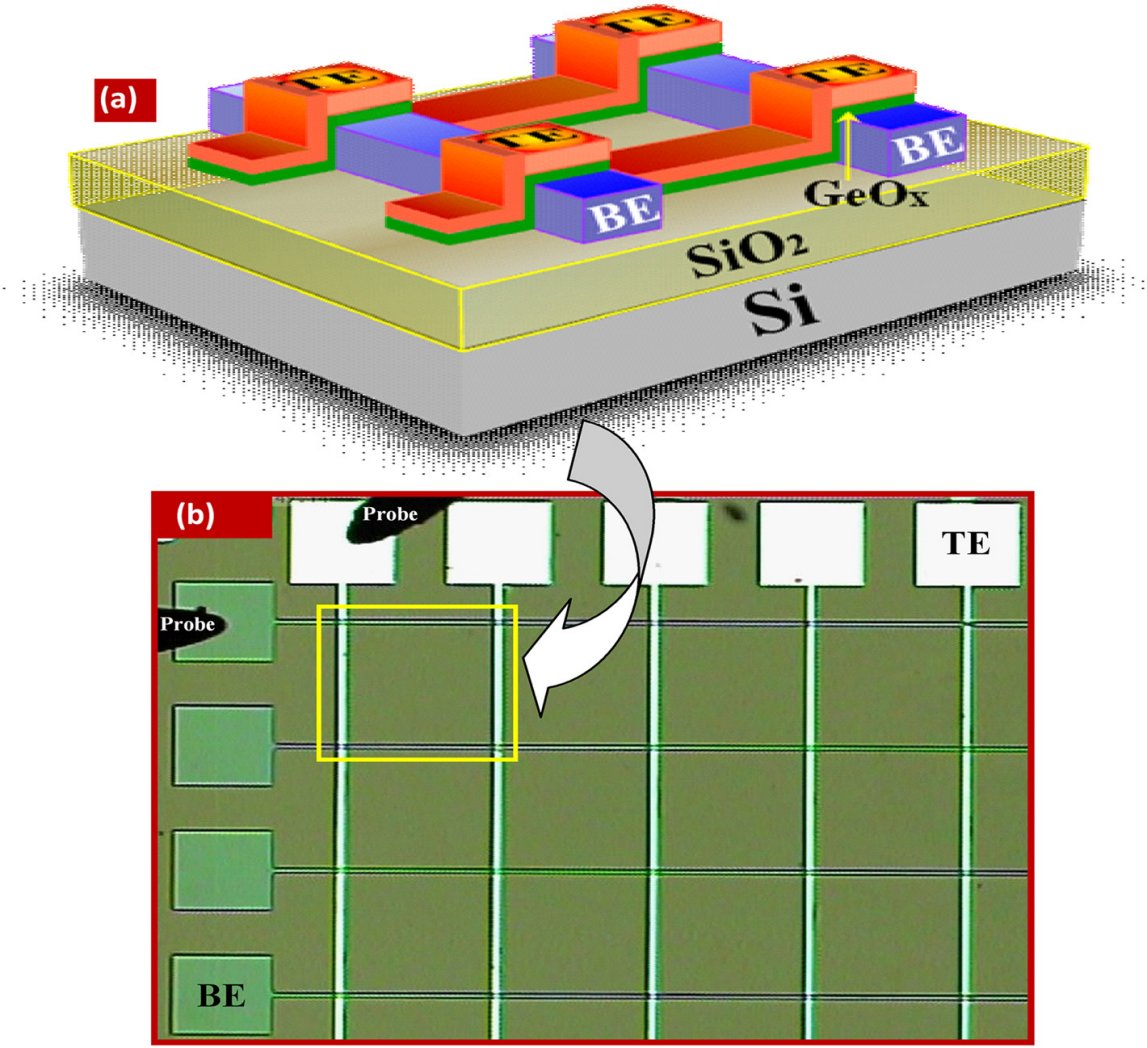
**Schematic illustration and optical image of the Cu/GeO**_***x***_**/W cross-point memories. (a)** Schematic illustration and **(b)** optical image of our fabricated cross-point memory devices. Active area of the cross-point memory is approximately 1 × 1 μm^2^. The thickness of the GeO_*x*_ solid electrolyte film is approximately 10 nm.

A silicon dioxide (SiO_2_) layer with a thickness of approximately 200 nm was grown by wet oxidation process on 4-in.p-Si wafers after the Radio Corporation of America (RCA) cleaning method. The horizontal furnace consisted of a quartz tube on a carrier made of quartz glass in the middle; the wafers were placed in the quartz tube. The temperature of the furnace was maintained at 900°C during the oxidation process. To avoid cracks or warping, the wafers were placed inside the furnace at 600°C. The furnace was heated slowly with a ramp rate of +13°C/min. During the oxidation process, hydrogen and oxygen gases were used with flow rates of 4 and 2.5 standard liters per minute (SLM), respectively. The oxidation time was 90 min. Then, W metal as a bottom electrode (BE) with a thickness of approximately 200 nm was deposited by radio frequency (RF) sputtering on SiO_2_/Si wafers. The deposition parameters of the W layer were shown in Table 1. Then, the BE was defined and patterned by standard photolithography and wet chemical etching processes. The following parameters were used for the photolithography process. The wafer is initially heated at 120°C for 10 min in the oven to drive off any moisture that may be present on the wafer surface. A liquid ‘adhesion promoter’ such as hexamethyldisilazane or HMDS was applied to promote adhesion of the photoresist to the wafer. A spin coater was used to coat the HMDS on the wafer. The spin coating was run at initially 3,000 rpm for 10 s and then 5,000 rpm for 20 s. Following the same process, an AZ6112 positive photoresist (AZ Electronic Materials, Branchburg, NJ, USA) was spun on the wafer to create the pattern. The photoresist-coated wafer was then prebaked to drive off excess photoresist solvent at 90°C for 2 min. After prebaking, the sample was placed on a vacuum substrate of an optical lithography system (ABM Sales Service, San Jose, CA, USA). Then, mask 1 was placed over the sample. The photoresist was exposed to ultraviolet (UV) light for 4 s. Before developing, a postexposure bake (PEB) was performed at 90°C for 1 min to reduce the standing wave phenomena caused by the destructive and constructive interference patterns of the incident light. The wafer was immersed into the AZ330 developer (AZ Electronic Materials) for 15 s to remove the exposed photoresist and then rinsed by deionized (DI) water. The resulting wafer was then ‘hard-baked’ to harden the final resist at 120°C for 15 min. The wet chemical etching process was used to etch the uncovered W metal layer and form the W BE. A commercially available tungsten etchant (Sigma-Aldrich) was used, and the wafer was dipped into the solution for 2 to 3 min. The same photolithography process was repeated to design the 1 × 1 to 10 × 10 arrays. To pattern the switching material and TE, mask 2 was placed over the samples using a mask aligner. After the masking process, the GeO_*x*_ switching material with a thickness of approximately 10 nm was deposited by the same RF sputtering system. Following this, Cu as a TE with a thickness of approximately 40 nm was deposited using a thermal evaporator. Then, the aluminum (Al) layer with a thickness of approximately 160 nm was deposited *in situ* by the thermal evaporator. The deposition conditions of GeO_*x*_, Cu, and Al were shown in Table 1. Finally, a lift-off process was performed to get the final Al/Cu/GeO_*x*_/W (device S1) memory device, i.e., called Cu/GeO_*x*_/W structure hereafter. Similarly, an Al/GeO_*x*_/W (device S2) memory device without a Cu layer was also prepared for comparison. Table 2 shows the structures of the fabricated memory devices. A schematic illustration of the fabricated GeO_*x*_-based cross-point memory device is shown in Figure 1a. The GeO_*x*_ solid electrolyte is sandwiched between Cu or Al TE and W BE. An optical micrograph (OM) of 4 × 5 cross-points is shown clearly in Figure 1b. All cross-points are clearly observed.

**Table 1:** Deposition parameters of different materials

**Materials**	**Target/granules**	**Methods**	**Vacuum (Torr)**	**Ar gas (SCCM)**	**Power (Watt)**	**Deposition rate**
W	W target	RF sputtering	1 × 10^-5^	25	150	12 nm/min
GeO_*x*_	Ge target	RF sputtering	2 × 10^-5^	25	50	5.3 nm/min
Cu	Cu granules	Thermal evaporator	8 × 10^-6^	-	-	2-3 Å/s
Al	Al granules	Thermal evaporator	8 × 10^-6^	-	-	2-3 Å/s

**Table 2:** Structures of the cross-point resistive switching memory devices

**Devices**	**BE ~ 200 nm**	**Switching layer (10 nm)**	**TE**	
			**Cu ~ 40 nm**	**Al ~ 160 nm**
S1	W	GeO_*x*_	**√**	**√**
S2	W	GeO_*x*_	**×**	**√**

The cross-point structure and thicknesses of all materials were evaluated from a HRTEM image. HRTEM was carried out using a FEI Tecnai (Hillsboro, OR, USA) G2 F-20 field emission system. Memory characteristics were measured using an HP4156C semiconductor parameter analyzer (Agilent Technologies, Santa Clara, CA, USA). For electrical measurements, the bias was applied to the TE while the W BE was grounded.

## Results and discussion

**Figure 2:**
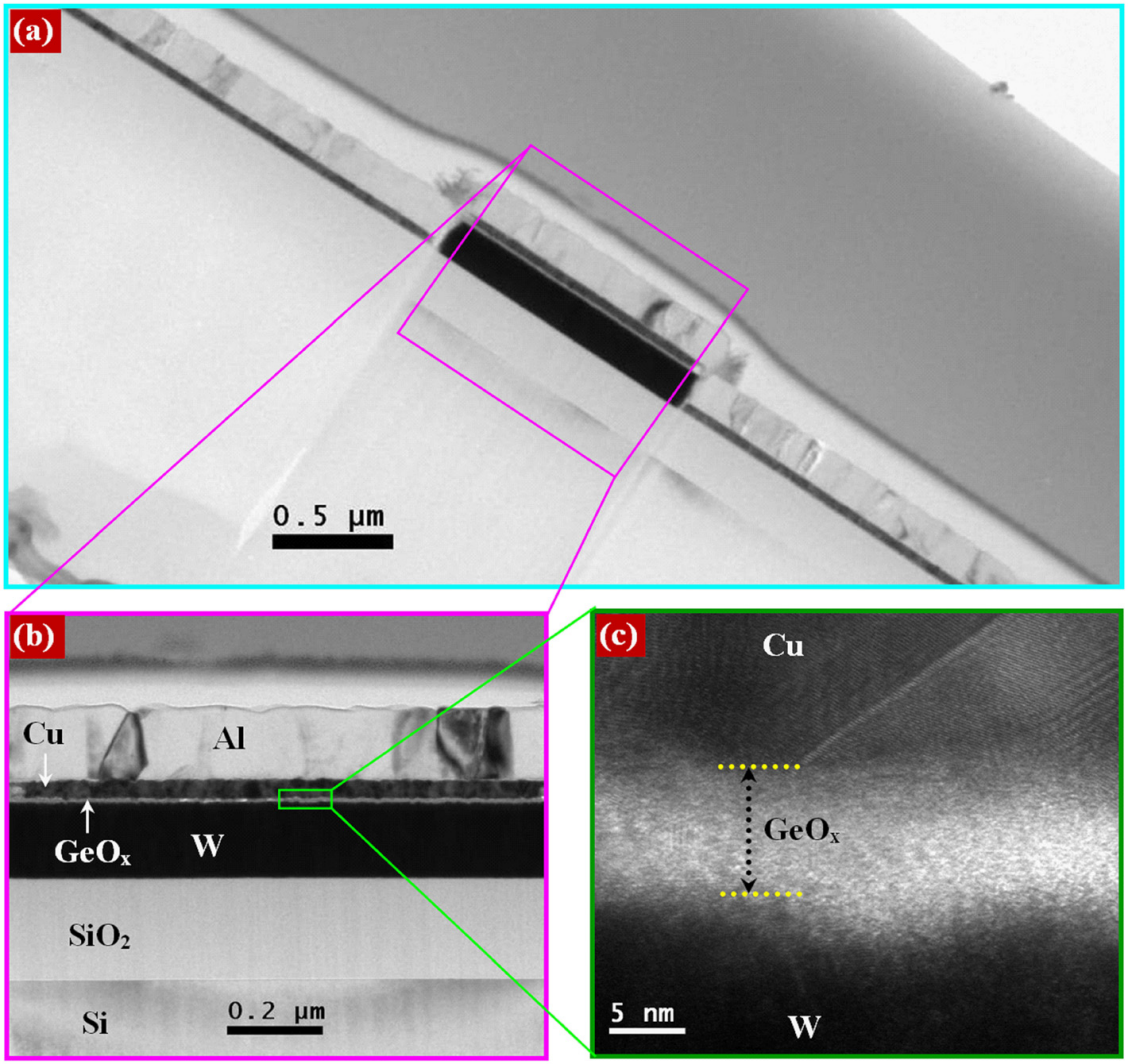
**TEM images of the cross-point memories using Cu electrode. (a)** TEM image of a Cu/GeO_*x*_/W cross-point memory. HRTEM image with scale bars of **(b)** 0.2 μm and **(c)** 5 nm. Films deposited layer by layer are clearly observed by HRTEM imaging.

**Figure 3:**
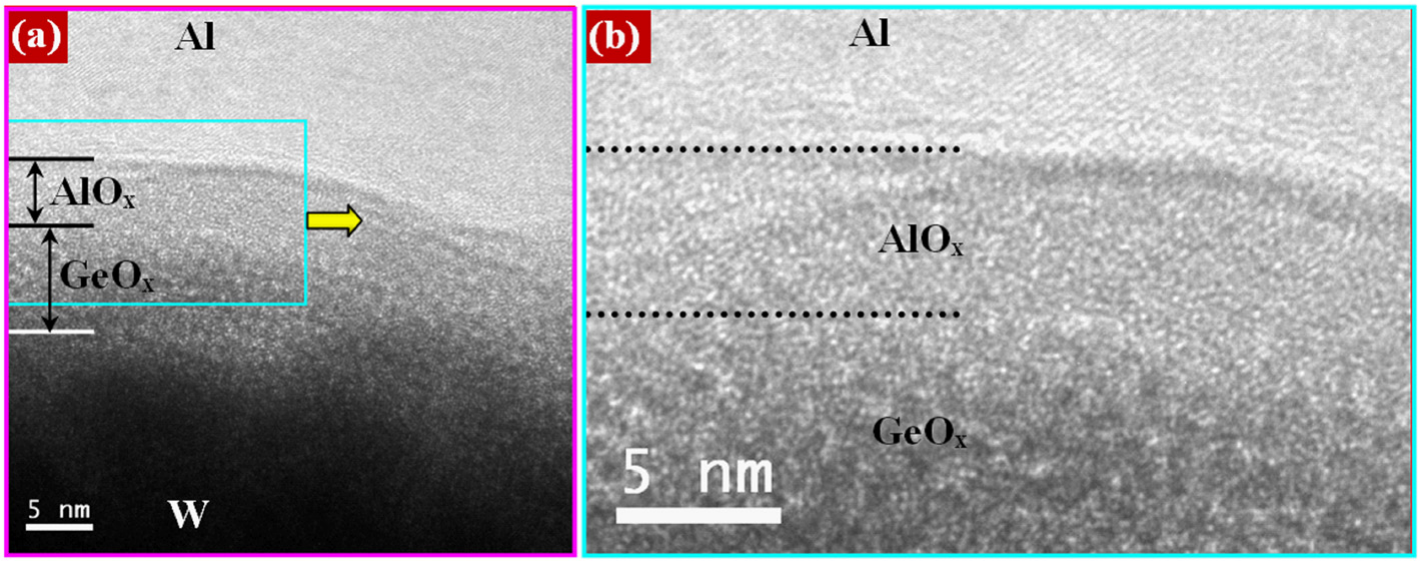
**TEM images of the device using Al electrode. (a)** HRTEM image of an Al/GeO_*x*_/W cross-point memory. **(b)** Formation of an AlO_*x*_ film with a thickness of approximately 5 nm at the Al/GeO_*x*_ interface is observed.

Figure 2 shows the TEM image of the Cu/GeO_*x*_/W structure (device S1). The area of the cross-point is approximately 1.2 × 1.2 μm^2^ (Figure 2a). Films deposited layer by layer are clearly observed in the HRTEM image, as shown in Figure 2b. The thickness of the SiO_2_ layer is approximately 200 nm. The thicknesses of W, Cu, and Al metals are approximately 180, 38, and 160 nm, respectively. The thickness of the GeO_*x*_ solid electrolyte is approximately 8 nm, as shown in Figure 2c. The formation of a thin (2 to 3 nm) WO_*x*_ layer is observed at the GeO_*x*_/W interface. The HRTEM image of the Al/GeO_*x*_/W cross-point memory devices is also shown in Figure 3a. It is interesting to note that the AlO_*x*_ layer with a thickness of approximately 5 nm at the Al/GeO_*x*_ interface is observed (Figure 3b). The Gibbs free energies of the Al_2_O_3_, GeO_2_, CuO, and Cu_2_O films are -1,582, -518.8, -129.7, and -149 kJ/mol at 300 K, respectively [43]. Therefore, the formation of AlO_*x*_ at the Al/GeO_*x*_ interface will be the easiest as compared to those of other materials. For example, the AlO_*x*_ layer at the Al/Ta_2_O_5_ interface was also observed even though the Al layer was deposited on a Ta_2_O_5_ film (not shown here). This suggests that Al is a metal reactive with oxygen, and it is hard to control the reaction at the Al/oxide interface. However, the AlO_*x*_ film will have more defects, which may have resistive switching phenomena. The resistive switching memory characteristics using Cu and Al top electrodes on GeO_*x*_/W cross-point memories are discussed below.

**Figure 4:**
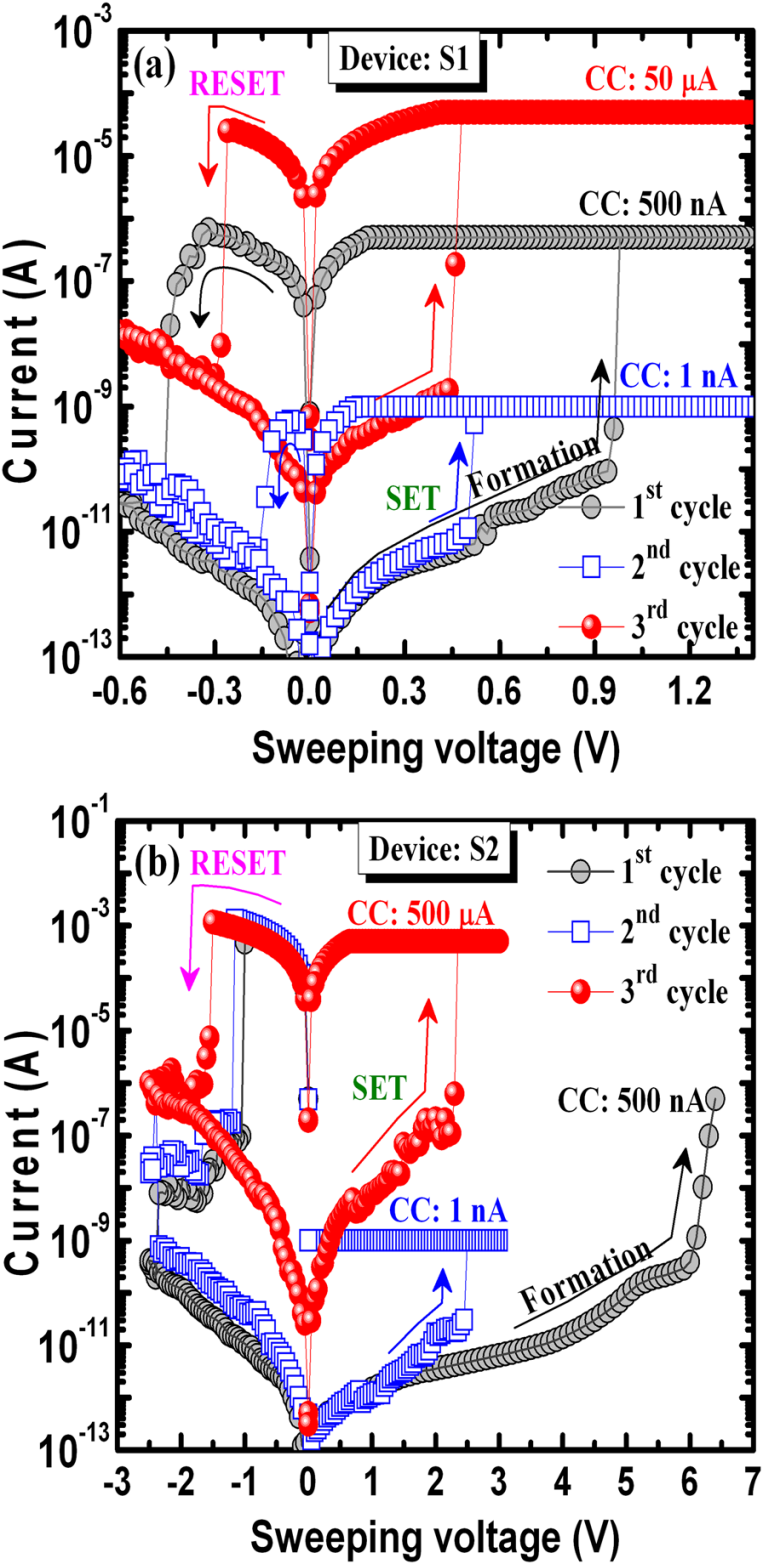
**Bipolar resistive switching characteristics. (a)** Typical *I*-*V* characteristics of Cu/GeO_*x*_/W and **(b)** Al/GeO_*x*_/W cross-point memories.

**Figure 5:**
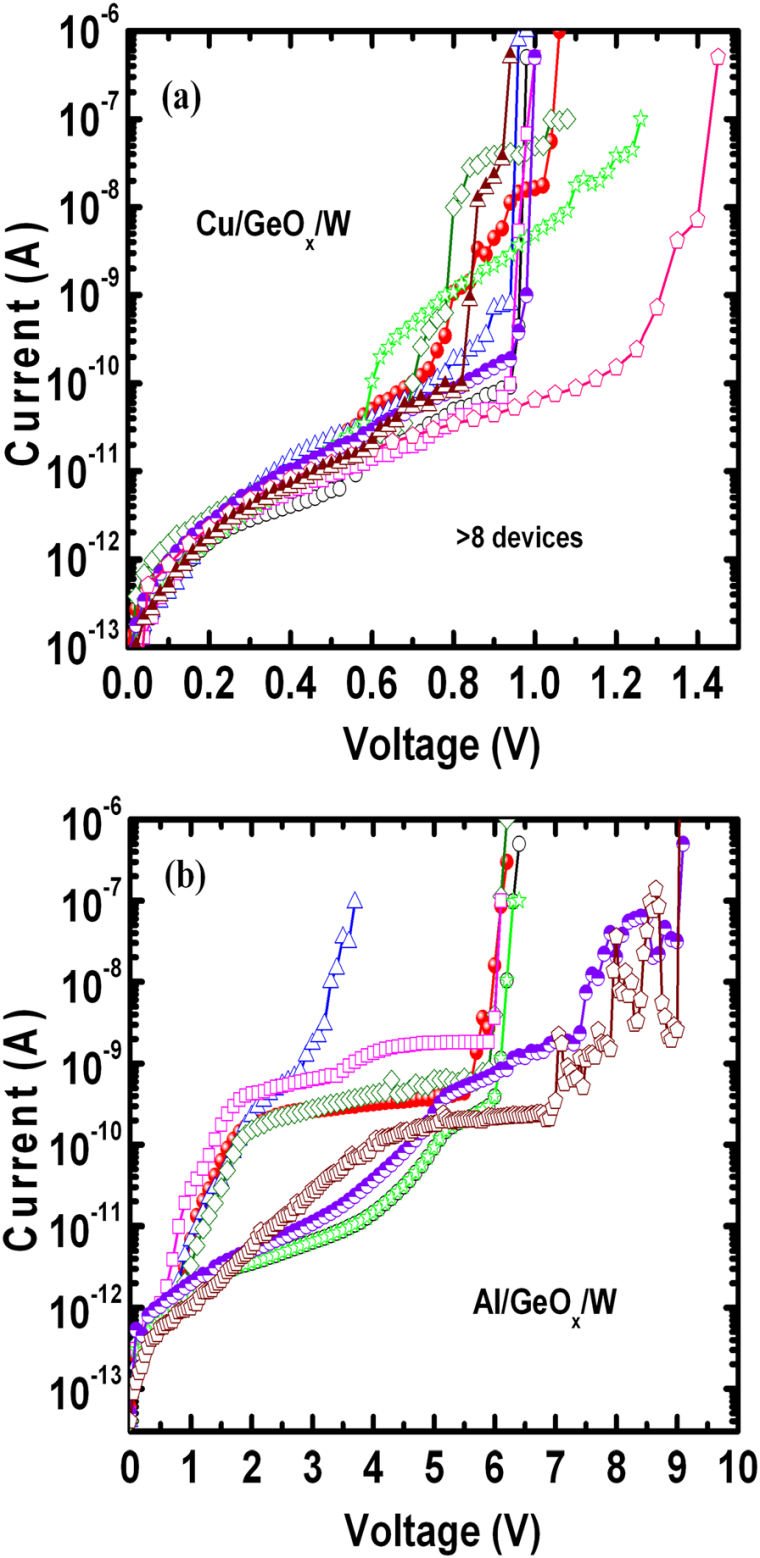
**Current–voltage characteristics.***I*-*V* measurements of pristine **(a)** Cu/GeO_*x*_/W (S1) and **(b)** Al/GeO_*x*_/W (S2) devices. A high formation voltage is needed for Al TE. More than eight devices were measured randomly.

Typical *I*-*V* hysteresis with CCs of 1 nA to 50 μA when using the Cu/GeO_*x*_/W cross-point memory is shown in Figure 4a. Initially, all memory devices were in high-resistance state (HRS), and positive sweeping voltage was applied. A slightly high voltage of approximately 1 V is necessary to switch the memory device from HRS to low-resistance state (LRS) under a CC of 500 nA, which is shown in the first cycle. This will form a Cu filament in the GeO_*x*_ solid electrolyte. After the formation process, the device shows normal bipolar resistive switching behavior. The memory device can be operated at a low CC of 1 nA, and a Cu cylindrical-type filament can be expected to form because the currents at HRS are the same after RESET operation for CCs of 1 to 500 nA [33]. A current change at HRS (approximately 1 pA to 1 nA at 0.1 V) is observed at a CC of 50 μA. At a higher CC of 50 μA, the filament diameter increased and the shape of the filament will be conical type [27]. This implies that the Cu filament remains at the GeO_*x*_/W interface after RESET operation. On the other hand, a high formation voltage of approximately 6 V is needed for the Al TE, as shown in the first cycle (Figure 4b). In this case, the memory device can be operated at a low CC of 1 nA, but a high RESET current of >1 mA is needed to rupture the conducting filaments. A current change at HRS is observed at a high CC of 500 μA owing to the remaining filament even with a higher RESET current of >1 mA. *I*-*V* measurements for pristine devices S1 and S2 are shown in Figure 5a,b. The average leakage currents at 0.1 V of the S2 devices are higher than those of the S1 devices (4.4 pA versus 0.4 pA) owing to the formation of the approximately 5-nm-thick AlO_*x*_ layer at the Al/GeO_*x*_ interface. The formation voltages for the S1 devices are 0.8 to 1.4 V, while they are 3 to 9 V for the S2 devices, which is due to the thicker switching material for the Al TE than the Cu TE (8 + 5 = 13 nm versus 8 nm). This is also beneficial to the Cu TE (device S1) than the Al TE (device S2).

**Figure 6:**
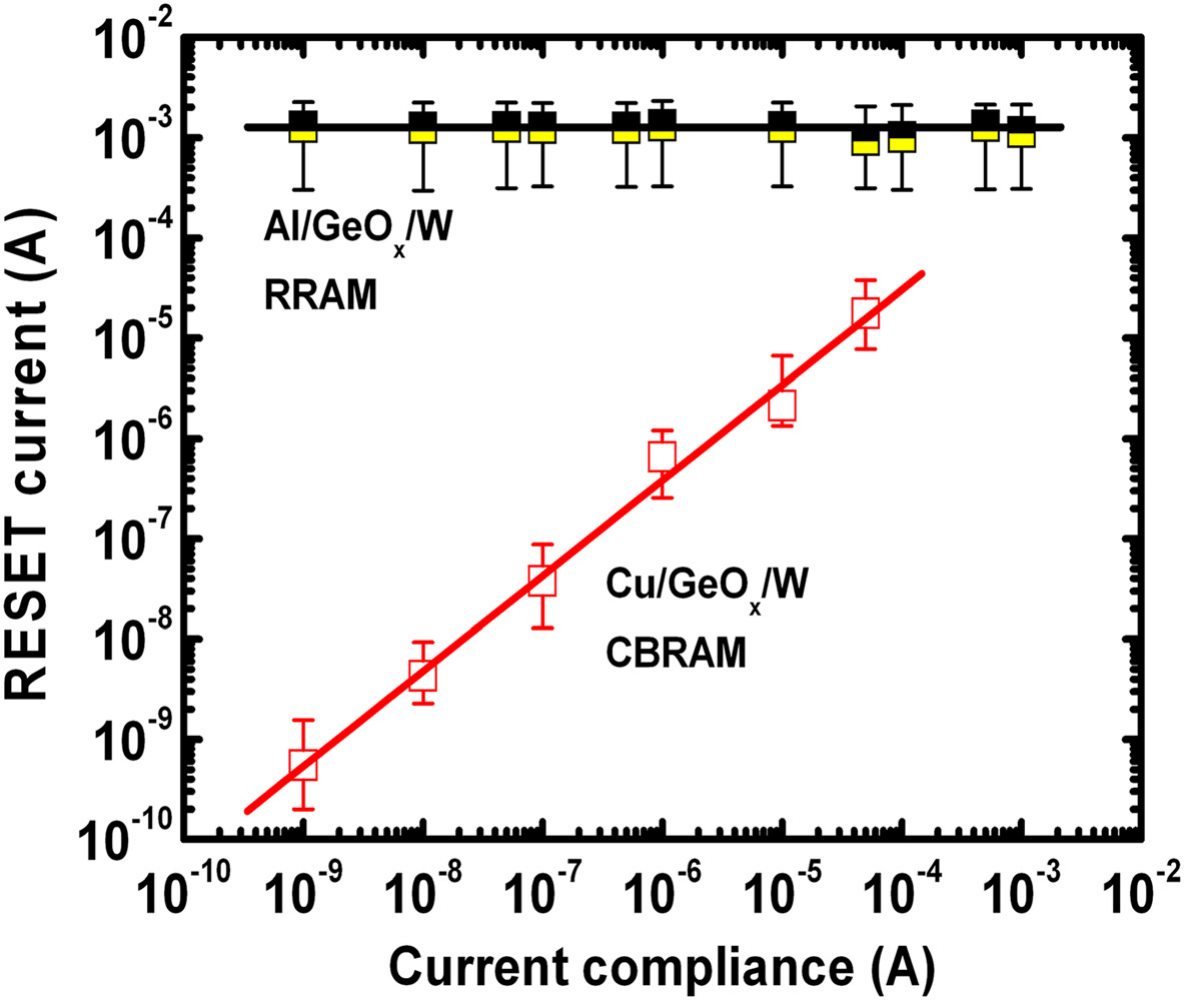
**Unipolar resistive switching characteristics.** Unipolar resistive switching characteristics of the Cu/GeO_*x*_/W cross-point memory device. A high resistance ratio of >10^8^ was also obtained using the cross-point architecture.

**Figure 7:**
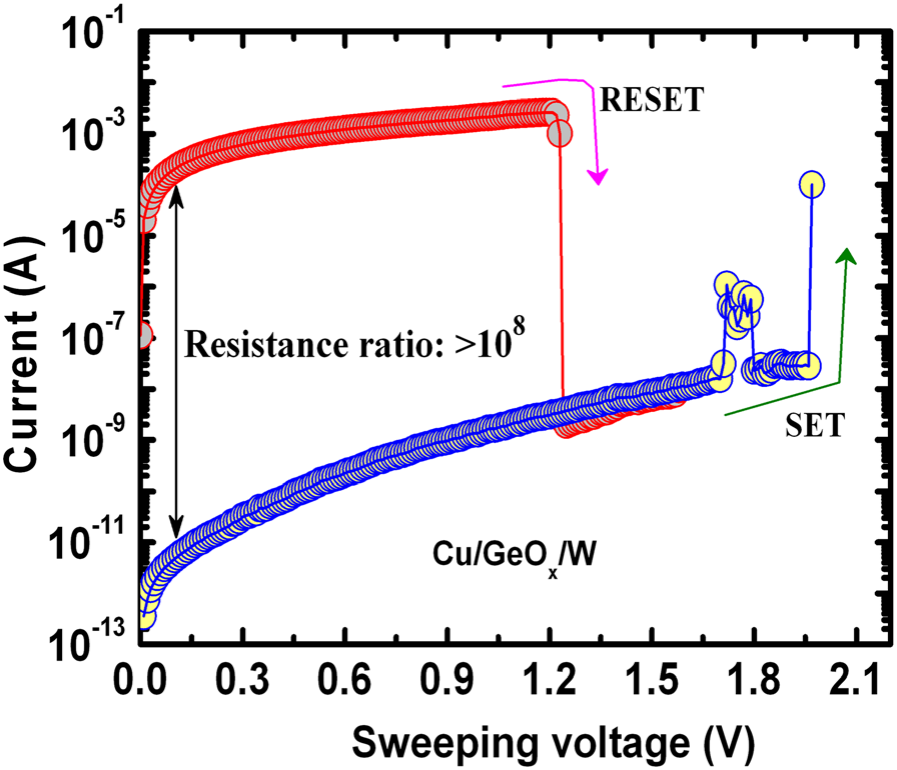
**RESET current scalability comparison with Cu and Al electrodes.** RESET currents versus CCs curve. The RESET current increases as the CCs for Cu TE increase; however, the RESET current is not scalable for Al TE because of the AlO_*x*_ formation at the Al/GeO_*x*_ interface.

Further, the RESET current is independent of CCs from 1 nA to 1 mA for the Al/GeO_*x*_/W cross-point memory device, as shown in Figure 6. This suggests that the RESET current scalability as well as device scaling is difficult for the Al TE devices, which form larger filament diameter (or many conducting filaments) even at a small CC of 1 nA. This is due to a strong current overshoot effect in the Al/GeO_*x*_/W cross-point memory devices. It is noted that the diameters of the conducting filaments are the same at all CCs from 1 nA to 2 mA, which is due to the defective AlO_*x*_ layer at the Al/GeO_*x*_ interface or unstable interface. A high RESET current of >20 mA was also reported by Kato et al. using Al TE [44]. Lin et al. [12] also reported a high RESET current for Al_2_O_3_-based resistive switching memory using a Ti/Al_2_O_3_/Pt structure. According to several reported results, using Al electrode or Al_2_O_3_-based resistive memory devices requires higher operation voltages as well as high RESET currents [12,44,45]; however, a few results were reported on low-current operation [6-8,14]. As we can see, the formation voltage of the Al/GeO_*x*_/W device is higher than that of the Cu/GeO_*x*_/W device. It seems that the parasitic capacitance [46] of the Al/GeO_*x*_/W device as well as the current overshoot effect is higher. Even if the SET voltage is lower, the RESET current is still very high or the same with the RESET current of formation. This suggests that the current overshoot effect is not due to the higher operation voltage but to the AlO_*x*_ formation at the Al/GeO_*x*_ interface or unstable interface. This is a very important difference between these Al and Cu TEs. An excellent scaling of the RESET current is observed for the Cu/GeO_*x*_/W cross-point memory devices with CCs from 1 nA to 50 μA. Furthermore, the RESET current is lower than the SET current, which proves no current overshoot effect even in the 1R configuration or no parasitic effect [46]. The formation and dissolution of Cu nanofilament under SET and RESET are responsible for the switching mechanism of the Cu/GeO_*x*_/W cross-point memory devices. The Cu ions will migrate through the defects into the GeO_*x*_ film and start to grow first at the GeO_*x*_/W BE under SET operation by reduction process (Cu^*z*+^ + ze^-^ → Cu^o^). The Cu nanofilament will start to dissolve at the Cu/GeO_*x*_ interface under RESET operation by oxidation process (Cu^o^ → Cu^*z*+^ + ze^-^). In the case of the Al/GeO_*x*_/W cross-point memory, oxygen vacancy filament formation and oxidation are responsible for the switching mechanism. When the applied bias voltage is higher than the SET voltage on the Al TE, the Ge-O bonds will break and O^2-^ ions as negative charge will migrate from the GeO_*x*_ layer towards the Al/GeO_*x*_ interface, resulting in an oxygen vacancy conducting filament formation. The RESET will occur when the applied negative bias on the Al TE is lower than the RESET voltage and the O^2-^ ions will migrate from the Al/AlO_*x*_ interface and oxidize the conducting filament. Due to the defective AlO_*x*_ layer formation at the Al/GeO_*x*_ interface and Joule heating, uncontrolled oxygen vacancy filament formation and oxidation by O^2-^ ion migration can be assumed under SET and RESET operations, which make reduction of the RESET current as well as scaling of the device difficult. This suggests that the Cu nanofilament diameter can be controlled by external CCs for the Cu/GeO_*x*_/W cross-point memories. In addition, unipolar resistive switching characteristics are also observed, as shown in Figure 7. In this case, the Cu filament is formed under SET and the filament is dissolved by Joule heating under RESET. A high resistance ratio of 10^8^was obtained from unipolar switching. Guan et al. [47] have also reported a high resistance ratio of approximately 10^6^using a Cu/ZrO_2_:Cu/Pt structure. This suggests that our new Cu/GeO_*x*_/W cross-point memory is useful for future multilevel cell (MLC) applications.

**Figure 8:**
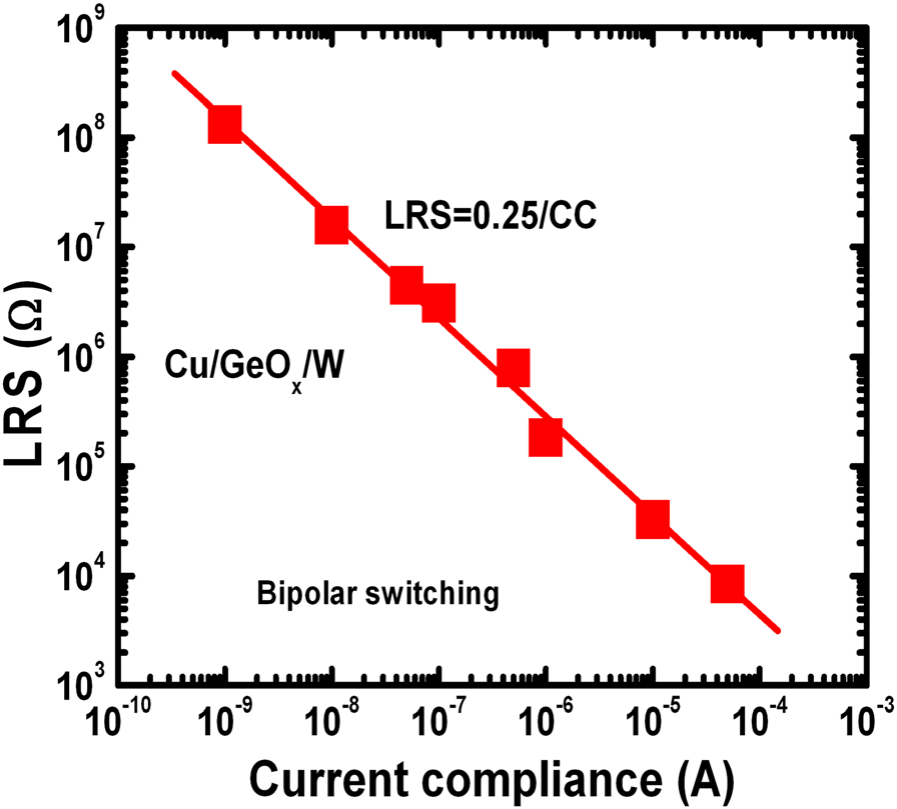
**LRS depends on CCs.** LRS versus CCs for the Cu/GeO_*x*_/W cross-point memory. LRS decreases with increasing CCs. The device can be operated with current as low as 1 nA.

Figure 8 shows the dependence of LRS on CCs ranging from 1 nA to 50 μA for the Cu/GeO_*x*_/W cross-point memories. The LRSs decreased linearly with increase of the CCs from 1 nA to 50 μA, which is applicable for MLC operation. By changing CCs (1 nA to few microamperes), more than four orders of magnitude of the LRS is shifted over the same range. If we consider that 3 resistance states per decade can be distinguished [3], the resistive memory using the Cu/GeO_*x*_/W structure will allow at least 12 states for the storage. The relationship between LRS and CC is related to the following equation:

(1) $$\mathrm{LRS}\;=\;\frac{0.25}{\mathrm{CC}}$$

From Equation 1, the average LRS is 0.251/CC, which is close to the reported value of 0.250/CC for metallic filament [33,48]. Therefore, the CBRAM device can be designed easily for low-power MLC operation.

**Figure 9:**
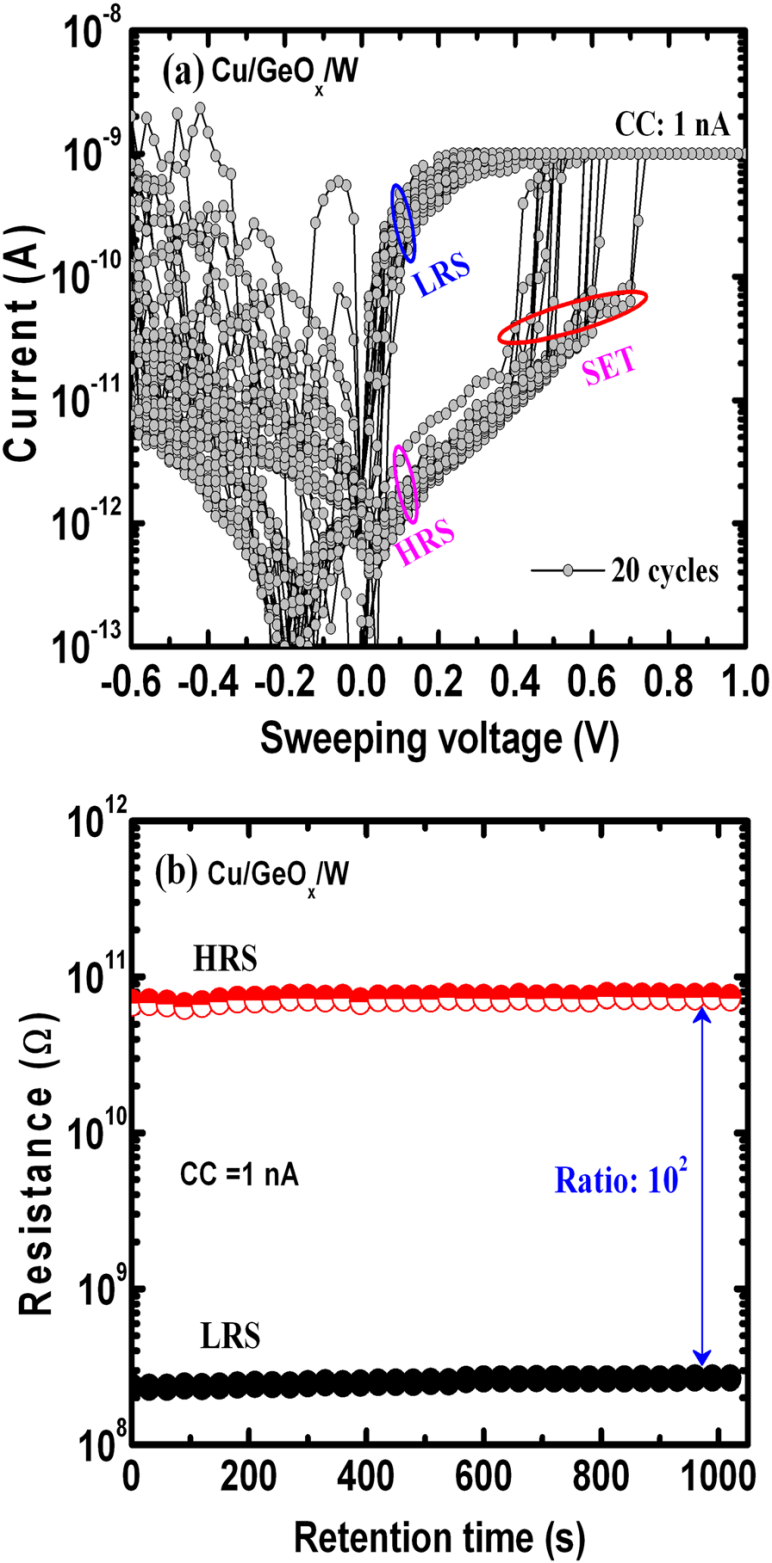
**Switching cycles and data retention. (a)** Repeatable switching cycles and **(b)** data retention of the Cu/GeO_*x*_/W cross-point memory devices under a low CC of 1 nA.

Figure 9a shows repeatable 20 DC switching cycles at a low CC of 1 nA. The SET voltages are varied from 0.4 to 0.8 V, and the RESET current increased after few cycles, which confirms a filament formation after few cycles [20,30]. The data retention of approximately 10^3^ s is also observed under a low operation current of 1 nA (Figure 9b). The resistance ratio is approximately 10^2^. Further study is needed to improve the cross-point resistive switching memory characteristics under low-current operation. In addition, the read pulse endurances of LRS and HRS are more than 10^5^ cycles with a large resistance ratio of >10^4^, and both resistance states are very stable without significant resistance variation for a retention test of more than 10^4^ s under a CC of 50 μA (not shown here), which can be applicable for future low-power high-density nonvolatile memory applications.

## Conclusions

Resistive switching memory characteristics using Cu and Al TEs on the GeO_*x*_/W cross-point memory devices have been compared. Improved memory characteristics of the Cu/GeO_*x*_/W structures under low current varying from 1 nA to 50 μA and a low voltage operation of ±2 V are observed as compared to those of the Al/GeO_*x*_/W structures. These cross-point memory structures are observed by HRTEM. The formation of AlO_*x*_ layer with a thickness of approximately 5 nm at the Al/GeO_*x*_ interface is observed, which is unstable to control the resistive switching phenomena. The RESET current scalability is observed for Cu TE, while it is high (>1 mA) and independent for the Al TE with CCs varying from 1 nA to 500 μA. Superior resistive switching memory performances in terms of high resistance ratio (10^2^ to 10^4^ under bipolar and approximately 10^8^ under unipolar modes), long pulse endurance of >10^5^ cycles under a CC of 50 μA, and good scalability potential are observed for the Cu/GeO_*x*_/W cross-point memory devices. Repeatable switching cycles and data retention of 10^3^ s are also observed under a low CC of 1 nA. This study is important for high-density low-power 3D architecture in the future.

## Competing interests

The authors declare that they have no competing interests.

## Authors’ contributions

SZR fabricated and measured the cross-point memory devices under the instruction of SM. SM arranged and finalized the manuscript. Both authors contributed to the preparation and revision of the manuscript and approved it for publication.

## References

[B1] Sawa A Resistive switching in transition metal oxides Mater Today 2008 11 28

[B2] Kim DC Seo S Ahn SE Suh DS Lee MJ Park BH Yoo IK Baek IG Kim HJ Yim EK Lee JE Park SO Kim HS Chung UI Moon JT Ryu BI Electrical observations of filamentary conductions for the resistive memory switching in NiO films Appl Phys Lett 2006 88 202102 10.1063/1.2204649

[B3] Waser R Aono M Nanoionics-based resistive switching memories Nat Mater 2007 6 833 10.1038/nmat2023 17972938

[B4] Sun X Li G Chen L Shi Z Zhang W Bipolar resistance switching characteristics with opposite polarity of Au/SrTiO_3_/Ti memory cells Nanoscale Res Lett 2011 6 599 10.1186/1556-276X-6-599 22107926 PMC3260421

[B5] Ninomiya T Wei Z Muraoka S Yasuhara R Katayama K Takagi T Conductive filament scaling of TaO_x_ bipolar ReRAM for improving data retention under low operation current IEEE Trans Electron Devices 2013 60 1384

[B6] Lee MJ Lee CB Lee D Lee SR Chang M Hur JH Kim YB Kim CJ Seo DH Seo S A fast, high-endurance and scalable non-volatile memory device made from asymmetric Ta_2_O_5-x_/TaO_2-x_ bilayer structures Nat Mater 2011 10 625 10.1038/nmat3070 21743450

[B7] Prakash A Maikap S Chiu H-C Tien T-C Lai C-S Enhanced resistive switching memory characteristics and mechanism using a Ti nanolayer at the W/TaO_x_ interface Nanoscale Res Lett 2013 8 288 10.1186/1556-276X-8-288 PMC3853228 24229327

[B8] Prakash A Jana D Maikap S TaO_x_-based resistive switching memories: prospective and challenges Nanoscale Res Lett 2013 8 418 10.1186/1556-276X-8-418 24107610 PMC4124699

[B9] Chen YS Lee HY Chen PS Wu TY Wang CC Tzeng PJ Chen F Tsai MJ Lien C An ultrathin forming-free HfO_x_ resistance memory with excellent electrical performance IEEE Electron Device Lett 2010 31 1473

[B10] Chen YY Goux L Clima S Govoreanu B Degraeve R Kar GS Fantini A Groeseneken G Wouters DJ Jurczak M Endurance/retention trade-off on HfO_2_/metal cap 1T1R bipolar RRAM IEEE Trans Electron Devices 2013 60 1114

[B11] Kwon DH Kim KM Jang JH Jeon JM Lee MH Kim GH Li XS Park GS Lee B Han S Kim M Hwang CS Atomic structure of conducting nanofilaments in TiO_2_ resistive switching memory Nat Nanotechnol 2010 5 148 10.1038/nnano.2009.456 20081847

[B12] Lin CY Wu CY Wu CY Lee TC Yang FL Hu C Tseng TY Effect of top electrode material on resistive switching properties of ZrO_2_film memory devices IEEE Electron Device Lett 2007 28 366

[B13] Zhang T Zhang X Ding L Zhang W Study on resistance switching properties of Na_0.5_Bi_0.5_TiO_3_thin films using impedance spectroscopy Nanoscale Res Lett 2009 4 1309 10.1007/s11671-009-9397-4 20628453 PMC2893894

[B14] Wu Y Lee B Wong HSP Al_2_O_3_-based RRAM using atomic layer deposition (ALD) with 1-μA RESET current IEEE Electron Device Lett 2010 31 1449

[B15] Banerjee W Maikap S Lai CS Chen YY Tien TC Lee HY Chen WS Chen FT Kao MJ Tsai MJ Yang JR Formation polarity dependent improved resistive switching memory characteristics using nanoscale (1.3 nm) core-shell IrO_x_ nano-dots Nanoscale Res Lett 2012 7 194 10.1186/1556-276X-7-194 22439604 PMC3338378

[B16] Prakash A Maikap S Banerjee W Jana D Lai CS Impact of electrically formed interfacial layer and improved memory characteristics of IrO_x_/high-κ_x_/W structures containing AlO_x_, GdO_x_, HfO_x_, and TaO_x_ switching materials Nanoscale Res Lett 2013 8 379 10.1186/1556-276X-8-379 24011235 PMC3848679

[B17] Kund M Beitel G Pinnow CU Röhr T Schumann J Symanczyk R Ufert KD Müller G Conductive bridging RAM (CBRAM): an emerging non-volatile memory technology scalable to sub 20 nm IEEE International Electron Devices Meeting. IEDM Technical Digest: 5–7 December 2005 2005 Washington, DC: Piscataway: IEEE 754 757

[B18] Rahaman SZ Maikap S Chiu HC Lin CH Wu TY Chen YS Tzeng PJ Chen F Kao MJ Tsai MJ Bipolar resistive switching memory using Cu metallic filament in Ge_0.4_Se_0.6_solid-electrolyte Electrochem Solid-State Lett 2010 13 H159 10.1149/1.3339449

[B19] Yu S Wong HSP Compact modeling of conducting-bridge random-access memory (CBRAM) IEEE Trans Electron Dev 2011 58 1352

[B20] Rahaman SZ Maikap S Das A Prakash A Wu YH Lai CS Tien TC Chen WS Lee HY Chen FT Tsai MJ Chang LB Enhanced nanoscale resistive memory characteristics and switching mechanism using high Ge content Ge_0.5_Se_0.5_ solid electrolyte Nanoscale Res Lett 2012 7 614 10.1186/1556-276X-7-614 23130908 PMC3524762

[B21] Jameson JR Gilbert N Koushan F Saenz J Wang J Hollmer S Kozicki MN One-dimensional model of the programming kinetics of conductive-bridge memory cells Appl Phys Lett 2011 99 063506 10.1063/1.3623485

[B22] Sakamoto T Lister K Banno N Hasegawa T Terabe K Aono M Electronic transport in Ta_2_O_5_ resistive switch Appl Phys Lett 2007 91 092110 10.1063/1.2777170

[B23] Liu Q Long S Lv H Wang W Niu J Huo Z Chen J Liu M Controllable growth of nanoscale conductive filaments in solid-electrolyte-based ReRAM by using a metal nanocrystal covered bottom electrode ACS Nano 2010 4 6162 10.1021/nn1017582 20853865

[B24] Liu Q Sun J Lv H Long S Yin K Wan N Li Y Sun L Liu M Real-time observation on dynamic growth/dissolution of conductive filaments in oxide-electrolyte-based ReRAM Adv Mater 1844 2012 24 10.1002/adma.201104104 22407902

[B25] Liu Q Long S Wang W Tanachutiwat S Li Y Wang Q Zhang M Huo Z Chen J Liu M Low-power and highly uniform switching in ZrO_2_-based ReRAM with a Cu nanocrystal insertion layer IEEE Electron Device Letters 2010 31 1299

[B26] Li Y Long S Lv H Liu Q Wang Y Zhang S Lian W Wang M Zhang K Xie H Liu S Liu M Improvement of resistive switching characteristics in ZrO_2_film by embedding a thin TiO_x_ layer Nanotechnology 2011 22 254028 10.1088/0957-4484/22/25/254028 21572216

[B27] Rahaman SZ Maikap S Chen WS Lee HY Chen FT Tien TC Tsai MJ Impact of TaO_x_ nanolayer at the GeSe_x_/W interface on resistive switching memory performance and investigation of Cu nanofilament J Appl Phys 2012 111 063710 10.1063/1.3696972

[B28] Nagata T Haemori M Yamashita Y Yoshikawa H Iwashita Y Kobayashi K Chikyow T Bias application hard x-ray photoelectron spectroscopy study of forming process of Cu/HfO_2_/Pt resistive random access memory structure Appl Phys Lett 2011 99 223517 10.1063/1.3664781

[B29] Goux L Opsomer K Degraeve R Muller R Detavernier C Wouters DJ Jurczak M Altimime L Kittl JA Influence of the Cu-Te composition and microstructure on the resistive switching of Cu-Te/Al_2_O_3_/Si cells Appl Phys Lett 2011 99 053502 10.1063/1.3621835

[B30] Rahaman SZ Maikap S Tien TC Lee HY Chen WS Chen F Kao MJ Tsai MJ Excellent resistive memory characteristics and switching mechanism using a Ti nanolayer at the Cu/TaO_x_ interface Nanoscale Res Lett 2012 7 345 10.1186/1556-276X-7-345 22734564 PMC3436867

[B31] Peng S Zhuge F Chen X Zhu X Hu B Pan L Chen B Li RW Mechanism for resistive switching in an oxide-based electrochemical metallization memory Appl Phys Lett 2012 100 072101 10.1063/1.3683523

[B32] Yang Y Gao P Gaba S Chang T Pan X Lu W Observation of conducting filament growth in nanoscale resistive memories Nat Commun 2012 3 1737 10.1038/ncomms1737 22415823

[B33] Rahaman SZ Maikap S Chen WS Lee HY Chen FT Kao MJ Tsai MJ Repeatable unipolar/bipolar resistive memory characteristics and switching mechanism using a Cu nanofilament in a GeO_x_ film Appl Phys Lett 2012 101 073106 10.1063/1.4745783 PMC3436867 22734564

[B34] Beynon J El-Samanoudy MM Memory phenomena in reactively-evaporated AlO_x_ and GeO_x_ thin films J Mater Sci Lett 1987 6 1447 10.1007/BF01689318

[B35] El-Samanoudy MM Beynon J Scanning electron microscopy and electron microprobe analysis of Au-GeO_x_-Cu and Au-AlO_x_-Cu sandwich structures J Mater Sci 1991 26 2431 10.1007/BF01130191

[B36] Cheng C Chin A Yeh F Stacked GeO/SrTiO_x_ resistive memory with ultralow resistance currents Appl Phys Lett 2011 98 052905 10.1063/1.3549689

[B37] Syu YE Chang TC Tsai CT Chang GW Tsai TM Chang KC Tai YH Tsai MJ Sze SM Improving resistance switching characteristics with SiGeO_x_/SiGeON double layer for nonvolatile memory applications Electrochem Solid State Lett 2011 14 H419 10.1149/1.3615823

[B38] Schindler C Guo X Besmehn A Waser R Resistive switching in Ge_0.3_Se_0.7_ films by means of copper ion migration Z Phys Chem 2007 221 1469 10.1524/zpch.2007.221.11-12.1469

[B39] Yang JJ Pickett MD Li X Ohlberg DAA Stewart DR Williams RS Memristive switching mechanism for metal/oxide/metal nanodevices Nat Nanotechnol 2008 3 429 10.1038/nnano.2008.160 18654568

[B40] Kügeler C Meier M Rosezin R Gilles S Waser R High density 3D memory architecture based on the resistive switching effect Solid-State Electron 2009 53 1287 10.1016/j.sse.2009.09.034

[B41] Borghetti J Snider GS Kuekes PJ Yang JJ Stewart DR Williams RS Memristive switches enable stateful logic operations via material implication Nature 2010 464 873 10.1038/nature08940 20376145

[B42] Xia Q Yang JJ Wu W Li X Williams RS Self-aligned memristor cross-point arrays fabricated with one nanoimprint lithography step Nano Lett 2010 10 2909 10.1021/nl1017157 20590084

[B43] Birks N Meier GH Pettit FS Introduction to the High Temperature Oxidation of Metals 2006 Cambridge: Cambridge University Press

[B44] Kato S Nigo S Lee JW Mihalik M Kitazawa H Kido G Transport properties of anodic porous alumina for ReRAM J Phys Conf Ser 2008 109 012017

[B45] Song J Inamdar AI Jang BU Jeon K Kim YS Jung K Kim Y Im H Jung W Kim H Effects of ultrathin Al layer insertion on resistive switching performance in an amorphous aluminum oxide resistive memory Appl Phys Express 2010 3 091101 10.1143/APEX.3.091101

[B46] Kinoshita K Tsunoda K Sato Y Noshiro H Yagaki S Aoki M Sugiyama Y Reduction in the reset current in a resistive random access memory consisting of NiO_x_ brought about by reducing a parasitic capacitance Appl Phy Lett 2008 93 033506 10.1063/1.2959065

[B47] Guan W Long S Liu Q Liu M Wang W Nonpolar nonvolatile resistive switching in Cu doped ZrO_2_ IEEE Electron Device Letters 2008 29 434

[B48] Kozicki MN Mitkova M Waser R Memory devices based on mass transport in solid electrolytes Nanotechnology 2008 Weinheim: Wiley 3

